# Image Processing for Smart Agriculture Applications Using Cloud-Fog Computing

**DOI:** 10.3390/s24185965

**Published:** 2024-09-14

**Authors:** Dušan Marković, Zoran Stamenković, Borislav Đorđević, Siniša Ranđić

**Affiliations:** 1 Faculty of Agronomy in Čačak, University of Kragujevac, Cara Dušana 34, 32102 Čačak, Serbia; 2 Institute of Computer Science, University of Potsdam, An der Bahn 2, 14476 Potsdam, Germany; 3 IHP—Leibniz-Institutfür innovative Mikroelektronik, ImTechnologiepark 25, 15236 Frankfurt, Germany; 4 Institute Mihailo Pupin, Volgina 15, 11060 Belgrade, Serbia; bora@impcomputers.com; 5 Faculty of Technical Sciences Čačak, University of Kragujevac, Svetog Save 65, 32102 Čačak, Serbia; sinisa.randjic@ftn.kg.ac.rs

**Keywords:** image classification, cloud-fog computing, deep learning, agriculture application

## Abstract

The widespread use of IoT devices has led to the generation of a huge amount of data and driven the need for analytical solutions in many areas of human activities, such as the field of smart agriculture. Continuous monitoring of crop growth stages enables timely interventions, such as control of weeds and plant diseases, as well as pest control, ensuring optimal development. Decision-making systems in smart agriculture involve image analysis with the potential to increase productivity, efficiency and sustainability. By applying Convolutional Neural Networks (CNNs), state recognition and classification can be performed based on images from specific locations. Thus, we have developed a solution for early problem detection and resource management optimization. The main concept of the proposed solution relies on a direct connection between Cloud and Edge devices, which is achieved through Fog computing. The goal of our work is creation of a deep learning model for image classification that can be optimized and adapted for implementation on devices with limited hardware resources at the level of Fog computing. This could increase the importance of image processing in the reduction of agricultural operating costs and manual labor. As a result of the off-load data processing at Edge and Fog devices, the system responsiveness can be improved, the costs associated with data transmission and storage can be reduced, and the overall system reliability and security can be increased. The proposed solution can choose classification algorithms to find a trade-off between size and accuracy of the model optimized for devices with limited hardware resources. After testing our model for tomato disease classification compiled for execution on FPGA, it was found that the decrease in test accuracy is as small as 0.83% (from 96.29% to 95.46%).

## 1. Introduction

The amount of data generated by IoT devices can vary widely depending on the number of devices deployed, their sensors, and the frequency of data collection. The number of IoT devices worldwide is expected to increase in coming years, and therefore vast amounts of data will be generated. IoT data is used for real-time monitoring, predictive maintenance, healthcare applications, smart agriculture, smart cities, and more. Managing and processing large volumes of IoT data poses challenges related to data storage, security, real-time analytics, and bandwidth constraints. IoT devices collectively generate massive amounts of data that contribute to the growth of big data and drive the need for scalable data management and analytics solutions in various industries.

The development of IoT applications where complete data processing is performed in the Cloud can lead to a large network load and delay in returning processing results. Therefore, IoT applications that have requirements for delay-sensitive services and corresponding energy efficiency must be taken into consideration, and that does not only imply a Cloud-Only strategy. To support this type of service at the edge of the network, Cisco has introduced a new paradigm known as Fog computing. The advantages of its use are the reduction of energy consumption and data center load. If the Fog computing layer consists of computer devices with limited resources, it is necessary to allocate resources, considering requirements such as delay limits, application type, application priority or energy efficiency. An appropriate deployment strategy is chosen to determine where the task or part of the data processing application will be executed: on the End device, the Fog device, or the Cloud. Thus, the structure of the system and distribution of data flow is defined in relation to the requirements and main goals of IoT applications [1].

Image analysis via Convolutional Neural Networks (CNNs) has become increasingly important due to their ability to automatically learn and extract relevant features from images, leading to significant advancements across a wide range of applications. The depth of CNNs allows them to capture complex patterns and relationships within images, leading to highly accurate predictions. CNNs have found uses in many applications such as Image Classification, Object Detection, Image Segmentation, Facial Recognition and Medical Diagnosis [2].

CNNs can leverage modern hardware accelerators like GPUs and TPUs, making them highly efficient and capable of processing large datasets quickly.CNN architectures can be scaled to handle varying levels of complexity and computational resources, from mobile devices to large-scale data centers. Efficient CNN models can be deployed on Edge devices, enabling real-time inference without relying on Cloud computing, reducing latency, and enhancing privacy [3].

Image analysis in smart agriculture is increasingly important due to its potential to enhance productivity, sustainability, and efficiency in farming. By leveraging technologies like CNNs, Smart Agriculture can benefit from precise monitoring, early detection of issues, and optimized resource management.

The goal of our work is the realization of a model for image classification that can be optimized and adapted to be executed on devices with limited resources and at the level of Fog computing.

Data collection in the field of agronomy, using available equipment in the field, is not the same as some other fields with special laboratory conditions. In certain cases, with the most advanced laboratory equipment, for example, images of samples with very high resolutions can be obtained. In the field of agronomy, images are usually not characterized by high resolution and the number of images often depends on the season and conditions, so that sets of images of the expected size are not always obtained. The motivation of our research is to find an efficient way to select the appropriate classification model for a specific purpose and translate it into a version of the model that can be executed on devices with limited resources according to the concept of Fog computing, which would have a purpose in the field of agriculture.

The proposed solution makes the following contributions:

- Development of a comprehensive working and testing environment that selects an algorithm, creates and trains a deep learning model, and executes it on devices with limited hardware resources.
- A straightforward process is used to obtain a classification model accelerator without the need to deal with all details of FPGA implementation. This model could be effectively run on a Fog computing layer to achieve real-time responses, save energy and reduce network congestion towards the server.
- Indicators of the compromise between the size of the selected model and the achieved test accuracy of the model before and after translation of the model for Fog computing devices can be examined.
- The concept of the test environment can be transferred to another structure of Cloud-Fog computing in order to examine the performance of applications in the case when the classification model is executed on the Fog device or when its execution and data transfer is in the Cloud.

Obtaining results of the classification model in real time and without continuous dependence on the Cloud would enable state recognition from images in various cases in the field of Smart Agriculture, which mainly relies on devices with limited resources.

## 2. Fog Computing and Deep Learning

Fog computing represents a new paradigm that has extended the Cloud to the Edge of the network and is intended to be used with Cloud computing. The Fog computing level has similar characteristics to the Cloud, only with lower performance and closer to the data source, and the same applies to computer, network and storage resources. They are characterized by being able to perform some data processing near the IoT data source, without sending all the data to the Cloud. The nodes that form the Fog computing layer are connected to the corresponding IoT end devices. These end devices can be cameras, wearable devices, mobile phones, smart glasses, GPS devices, or various types of sensor-based devices. The architecture of Fog computing enables a different execution setting of IoT applications without the need to send data to the Cloud, which enables the fulfillment of requirements such as dynamic scalability, reduced latency and lower resource consumption in the Cloud [4].

IoT data processing on Edge devices and Fog layer involves handling data closer to the location where it is generated, which offers several advantages over traditional Cloud-centric approaches.

In the case of processing on Edge devices, low latency could be obtained by processing data locally, reducing the time it takes to transmit data to remote servers and receive responses. Also, there is a decrease of network load by reducing the amount of data sent to the Cloud due to processing or aggregating data locally, sending only relevant information. Privacy and security is improved by keeping sensitive data on local devices, minimizing exposure to potential security breaches during transmission. It is possible to perform offline operations independently of Cloud connectivity, maintaining functionality even when network connections are unreliable or unavailable. IoT applications with data processing on Edge devices enable real-time decision-making and responses based on immediate local data analysis.

**Figure 1:**
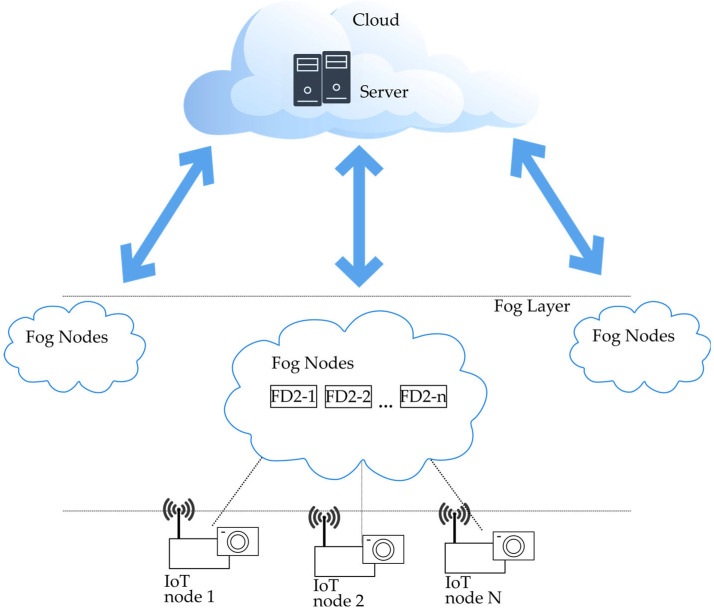
Cloud-Fog computing structure.

With the Fog layer formed, it is possible to conduct intermediate processing which may include aggregating and preprocessing data from multiple Edge devices before sending it to the Cloud, reducing the load on central servers (Figure 1). It also enables usage of Location-Based Services, since data would be processed within the proximity of the devices. A Fog layer with multiple nodes enables better scalability, enhancing system performance in large-scale deployments by distributing computational tasks across multiple Fog nodes. Fog devices are characterized by their integration with the Cloud and act as intermediaries between Edge devices and Cloud services, optimizing data transmission and storage.

Hardware acceleration for deep learning models on Edge devices involves using specialized hardware to improve performance and efficiency. Benefits of using acceleration can be seen through improved performance where hardware accelerators can significantly speed up inference times compared to running models solely on CPUs. Accelerators are designed to perform computations more efficiently, reducing power consumption on battery-powered devices. Also, accelerator support enables real-time processing of data without relying on Cloud services, enhancing responsiveness and privacy.

By using hardware accelerators, powerful deep learning models could be deployed on Edge devices for applications ranging from image and speech recognition to autonomous systems and IoT devices.

The application of artificial neural networks (ANNs) can be found in various business areas and industries, providing a number of services that are supported by high-performance Cloud computing and storage. On the other hand, there are requirements for applications that imply certain intelligence in embedded devices and Edge devices, such as autonomous systems or the Internet of Things (IoT). Although the computing capabilities of Edge devices have increased significantly in recent years, there are still challenges in executing certain neural network algorithms on devices with limited resources. The authors of [5] give a summary of hardware support at the network Edges for applications of machine learning techniques that require low power consumption.

A summary of research and challenges around Fog computing for IoT are given in [6], while a study on implementation of machine learning models in devices on the network Edge is presented in [7]. This article contains concepts, guidelines and future directions that may facilitate decentralization of machine learning to the network Edge devices, end devices, embedded systems and FPGA.

ANNs involve a large number of parameters and calculations, which require high energy consumption and memory usage. In order to transfer them to devices with limited resources, it is necessary to apply compression techniques in order to optimize the ANN models [8]. As neural networks have become more powerful, their depth and complexity have also made deployment on resource-constrained devices more challenging. Neural network quantization addresses this by reducing network precision, allowing for smaller and simpler networks that fit within hardware constraints. The authors of [9] survey recent quantization techniques and propose some future research directions.

CNNs work by progressively extracting higher-level features from the input image through a series of convolutional and pooling layers, followed by fully connected layers that perform the final classification or regression task. This hierarchical structure allows CNNs to learn complex patterns and relationships within the data, making them highly effective for various computer vision tasks [10].

## 3. Related Work

### 3.1. Image Classification and Smart Farming

The Internet of Things (IoT) has significantly advanced smart farming by facilitating extensive data collection for various agricultural processes. The authors of [11] examine the types of big data generated in smart farming, discuss its applications such as yield prediction and farm management, and explore key big data and machine learning techniques used in data analysis. The authors of [12] review the significant impact of the Internet of Things (IoT) and other smart technologies on agriculture, including Cloud computing, machine learning, and artificial intelligence. It discusses their applications in crop and animal production, post-harvesting processes, and considers the effects of climate change on agriculture.

Farmers are integrating modern technologies to optimize agricultural practices sustainably, leading to increased production. The authors of [13] present a detailed survey of ten key areas in smart farming, focusing on technologies like big data, machine learning, IoT, robotics, and others.

The authors of [14] explore the role of ICT technologies, including robotics, IoT devices, machine learning, and artificial intelligence, in advancing agriculture and addressing challenges in farming techniques. The paper reviews current applications, such as drones for crop observation and optimization, and identifies emerging research trends and issues in AI-driven agriculture.

The authors of [15] examine the transformative impact of digitization and automation on traditional farming practices through the use of computer vision and artificial intelligence (AI). The paper highlights how these technologies can enhance productivity and economic growth, and address global challenges like food security and sustainability.

Automation in image analysis using computer vision and deep learning enhances precision in field and yield mapping, achieving an average accuracy of 92.51% for crops like grapes, apples, and maize. The authors of [16] review various smart farming applications such as robot harvesting and weed detection, while also discussing limitations and future research needs in deep learning techniques.

Accurate plant disease identification is crucial for effective prevention in complex environments, and digital advancements in smart farming support this with data-driven methods.

Plant diseases significantly threaten crop production and food security, making accurate detection essential. While traditional methods like visual observation and lab tests have limitations, deep learning methods, particularly CNNs, have emerged as state-of-the-art solutions in plant disease classification. A review [17] highlights recent advancements in CNNs for plant disease detection.

Significant economic losses in agriculture occur due to crop infections, making early disease detection crucial, especially in a major rice-producing country like India. The authors of [18] propose a smart farming model that integrates machine learning and IoT to detect brown spot disease in rice using CNNs, achieving 97.701% accuracy.

The Internet of Things (IoT) enhances smart farming by enabling real-time data collection from agricultural fields, which, along with images and sensor data, allows for automated disease prediction using deep neural networks. Utilizing various sensors controlled by a Raspberry Pi 3 module, this system achieves 96% accuracy in leaf disease detection and classification through a CNN [19].

To enhance smart agriculture and increase plant productivity, continuous monitoring of plants from growth to harvest is crucial. Early detection and treatment of tomato diseases can significantly boost production, efficiency, and quality, leading to more affordable prices for consumers and preventing the farmers’ labor from being wasted.

The authors of [20] present an image-processing algorithm utilizing artificial neural networks to detect and monitor four tomato crop diseases. The algorithm categorizes images based on color, texture, and morphology, with morphology achieving the highest accuracy of 93%.

Tomatoes are a vital crop, with yield and quality heavily impacted by leaf diseases. Early identification and classification of these diseases are crucial for improving crop yields, and the use of CNN has shown promise in this area. In an experiment using Google Colab with a dataset of 3000 images, presented in [21], a CNN model was developed to classify tomato diseases with 98.49% accuracy.

The authors of [22] introduce a custom CNN model (CCNN) designed to classify tomato plant diseases, offering improved accuracy and reduced computational cost compared to existing models like AlexNet and VGG-16. The CCNN’s efficiency, with effective performance across 10 disease classes, makes it well-suited for smartphone-based disease detection.

The proposed technique in [23] utilizes CNN, specifically the Visual Geometry Group (VGG) model, to classify diseased and healthy leaves with high accuracy, achieving 98.40% accuracy for grapes and 95.71% for tomatoes.

The authors of [24] propose a compact CNN with only six layers for disease identification, which is computationally efficient and trained on the Plant Village tomato dataset. The proposed network outperforms well-known pre-trained deep networks, demonstrating that a smaller, less complex network can still deliver excellent results in tomato disease identification.

Improving food production through effective crop insect detection is crucial, as pest damage degrades crop quality. Traditional insect identification methods rely on skilled taxonomists and are less efficient compared to modern machine learning techniques. The authors of [25] present an insect pest detection algorithm that integrates foreground extraction and contour identification, achieving high classification accuracies of 91.5% and 90% with CNNs on various datasets.

Insect pests significantly impact crop yield and quality globally, making rapid and accurate monitoring essential for effective pest control. A review [26] examines the use of deep learning (DL) technology in smart pest monitoring (SPM), focusing on DL frameworks for insect pest classification and detection using field images.

The authors of [27] propose using a residual CNN with transfer learning for accurate pest identification, employing data augmentation techniques like random cropping and CutMix to enhance model robustness. The study shows that transfer learning significantly improves classification accuracy and reduces training time.

Excessive pesticide use can lead to harmful residues in the food chain. The work presented in [28] introduces a deep Convolutional Neural Network-based approach for detecting 102 common pest species. The final model, selected from 125 variations, is integrated into a mobile app that can classify pests through image capture or gallery selection, functioning both online and offline.

Inspecting sticky paper insect traps is crucial for effective pest management but is often labor-intensive and challenging. Recent research [29] presents an automated method using a CNN classifier to identify and count various insect pests from images. The developed algorithm achieved high performance with counting accuracies of 0.91 and 0.90, demonstrating its effectiveness for improving integrated pest management strategies in greenhouses.

Weeds significantly impact agricultural production, and full-coverage chemical herbicide spraying leads to environmental pollution and waste. Accurate weed detection and precise spraying are essential, requiring reliable identification of crops and weeds. A review [30] discusses traditional image-processing and deep learning-based methods for weed detection.

Weeds pose significant threats to crop yields, but advances in machine vision and image processing offer promising solutions for real-time weed detection in the field. A review [31] describes procedures for weed detection, including pre-processing, segmentation, feature extraction, and classification, focusing on techniques like color indices and machine learning.

The authors of [32] review DL-based techniques for weed detection and classification, focusing on data acquisition, dataset preparation, detection methods, and evaluation metrics. The paper highlights that supervised learning techniques, particularly fine-tuned pre-trained models, have achieved high accuracy when large labeled datasets are available.

Weed detection is crucial in precision farming, particularly within the IoT framework, as weeds cause significant crop losses. A recent paper [33] presents a vision-based weed detection system using deep learning models to effectively identify weeds in soybean plantations. Among the five models tested, including MobileNetV2, ResNet50, and three custom CNN models, a custom five-layer CNN achieved a high detection accuracy of 97.7%, with the lowest latency and memory usage when deployed on a Raspberry PI controller.

Precise weed recognition is essential for effective site-specific control in precision agriculture. The authors of [34] introduce a CNN-based graph convolutional network (GCN) approach for weed and crop recognition, which uses a GCN graph to integrate both labeled and unlabeled data for improved accuracy. The proposed GCN-ResNet-101 method achieved high recognition accuracies on four weed datasets and met real-time field control requirements.

### 3.2. CNN and Accelerators on Edge

The authors of [35] offer a comprehensive overview of recent advancements in CNNs, highlighting improvements in layer design, activation functions, loss functions, regularization, optimization techniques, and computational efficiency. They also discuss the wide array of applications where CNNs have demonstrated significant success, ranging from computer vision to speech and natural language processing.

CNNs have achieved significant success in computer vision and natural language processing, but their complexity and computational intensity limit practical applications, especially with increasing data dimensions. The authors of [36] review various network compression methods, focusing on pruning and quantization, to enhance CNN applicability in resource-constrained environments, and propose a novel framework to address the challenges of compressing large-scale CNNs.

In recent years, machine learning and deep learning have made significant advancements across various domains, but the large size of trained models poses challenges for deployment on resource-constrained devices like mobile phones and IoT devices. To enable real-time applications on such devices, it is crucial to compress and accelerate these models without compromising accuracy; this drives research into various techniques for model compression and acceleration. These actions are surveyed in [37] while addressing challenges and suggesting future research directions.

Deep learning has become integral to various services and applications, necessitating alternatives to Cloud-based training and inference due to latency concerns and the impending data overload from the Internet of Things. Edge computing emerges as a solution, but its limitations in power and resources require new, energy-efficient deep learning models and computing platforms. On this topic, the authors of [38] review key research directions in Edge computing deep learning algorithms.

High energy efficiency and re-configurability are promising features that suggest FPGA as a key platform for CNN hardware acceleration. The authors of [39] provide a comprehensive survey of techniques for implementing and optimizing CNN algorithms on FPGA, serving as a valuable resource for researchers in artificial intelligence, hardware architecture, and system design.

Widespread use of CNNs in various tasks, particularly on Edge devices with limited computing resources, is highlighted in [40]. Also, the authors propose FPGA-based custom computing architectures as a solution to enhance CNN inference performance, while maintaining accuracy.

The authors of [41] survey existing CNN-to-FPGA workflows, offering insights into their key characteristics such as supported applications, architectural choices, and performance. Also, they address major challenges and objectives introduced by the latest trends in CNN algorithmic research.

A survey [42] explores optimization techniques for vision CNNs on both algorithmic and hardware levels, which is essential for efficient implementation on resource-constrained devices, particularly FPGAs. This approach aims to address the challenge of fitting wider and deeper CNNs onto limited hardware resources by examining various optimization strategies.

An outline of the challenges of deploying deep learning (DL) models on Edge devices due to their limited resources, and the benefits of processing data directly on these devices to reduce latency and improve real-time decision-making, is given in [43]. To address these challenges, optimization techniques at both the hardware and software levels have been developed, focusing on novel DL architecture, algorithm design, optimization methods, algorithm-hardware co-design, and efficient accelerator design.

Image recognition using lightweight CNNs, which enable high-performance algorithms on resource-constrained devices, is explored in [44]. Theauthors review classical lightweight CNN models and recent image recognition techniques categorized into model compression, lightweight network optimization, and combining Transformer with lightweight networks.

The authors of [45] present a comprehensive review of neural network optimization technology based on FPGA, highlighting its importance and advantages in accelerating deep learning tasks.

An exploration of the implementation of CNNs on low-power embedded systems for use in a weeding robot to address the problem of weed control is presented in [46]. The article evaluates the technical feasibility of deploying CNNs on FPGAs, assesses optimization possibilities for both hardware and software, and investigates the performance of different networks on various hardware accelerators with diverse approaches.

The authors of [47] offer a thorough overview of recent advancements in computer vision algorithms and their hardware implementations. They focus on tasks like image classification, object detection, and image segmentation enabled by deep learning techniques. They review methods for optimizing and implementing these algorithms on various hardware accelerators such as GPU, FPGA, and emerging architectures, aiming to enable real-time and energy-efficient operations.

### 3.3. CNNs and Vision Transformers

Vision Transformers as modern methods have shown great performance in computer vision tasks such as image classification. Vision Transformers (ViTs) represent a type of deep learning model which applies the Transformer architecture previously designed and used for natural language processing (NLP). ViTs are applied in computer vision where image is treated as a sequence of patches, in the same way as words are treated in a sentence when dealing with NLP tasks [48].

ViTs could be used effectively to scale up with larger datasets and more computational resources. ViTs can capture long-range dependencies across the image, due to the self-attention mechanism. Using self-attention mechanisms allows them to consider the entire image at once, which can be very important in understanding complex visual scenes. Since ViTs treat an image as a sequence of patches, they could easily adapt to different image sizes by adjusting the number of patches, offering more flexibility in usage of various input dimensions.

Vision Transformers offer significant advantages over CNNs, especially in terms of scalability, flexibility and global context awareness. Usually, the training process of ViTs generally requires larger datasets and more computational resources.

Vision Transformers (ViTs) have been used in several cases where their strength comes to the fore. The authors of [49] introduce a dual-branch Transformer model that combines image patches of different sizes to improve feature representation. The model processes small and large patches separately and fuses them using attention mechanisms, with a cross-attention module that reduces computational complexity. Experiments show that this approach outperforms existing models, achieving better accuracy with a manageable increase in computational cost.

Recent advancements in multimodal data classification, such as combining hyperspectral images (HSI) and LiDAR, have improved remote sensing image accuracy. To address limitations of existing fusion methods, the authors of [50] propose a Modality Fusion Vision Transformer (MFViT) designed specifically for HSI and LiDAR fusion classification. These modules enhance the fusion of heterogeneous features and preserve spatial and spectral information, respectively. The model achieves superior classification accuracies of 99.91%, 99.59%, and 96.98% on three benchmark datasets, outperforming all existing methods and demonstrating its effectiveness and stability.

Another important application of automated image classification based on ViTs has high importance for decision-making for radiologists in detection of brain tumors. The authors of [51] explore the use of an ensemble of Vision Transformer (ViT) models for diagnosing brain tumors from T1-weighted (T1w) MRI images. They used four ViT models, the last of which (L/32) achieved the highest individual test accuracy of 98.2% at 384 × 384 resolution. Also, the ensemble of all four ViT models improved the accuracy to 98.7%, better than any individual model and showing potential for computer-aided diagnosis of brain tumor.

The authors of [52] explore the use of monocular depth estimation (MDE) in low-altitude drone flights, which are crucial for safety and monitoring operations. The study evaluates a state-of-the-art Vision Transformer (ViT) model, pre-trained on a large MDE dataset, comparing its performance against a classical fully convolutional network. The findings reveal that ViTs, after fine-tuning, can outperform convolutional models and are more robust to adversarial attacks, making them suitable for such critical applications.

Another modern technique used with great efficiency for image segmentation was presented in [53]. This paper addresses challenges in 3D image segmentation, which is crucial for improving segmentation accuracy in various fields like healthcare and military applications. To overcome the time complexity of Hidden Markov Models (HMMs), the authors propose a novel system that distributes the 3D segmentation process across multiple machines to speed up HMM training. The authors, through extensive experiments, demonstrate that the proposed approach is efficient and competitive with state-of-the-art methods in terms of security, segmentation accuracy, and execution time.

Also, there are applications of Vision Transformers in agriculture, such as the example presented in [54], where the authors propose GreenViT, a ViT-based technique for early plant disease detection. By dividing images into smaller patches and processing them with ViTs, GreenViT effectively overcomes CNN limitations.

Another study [55] also proposes a solution based on a ViT model to identify healthy and diseased plants. Additionally, a ViT-based Android app was developed, showing promise for large-scale smart agriculture applications and inspiring future research in the field.

The authors of [56] propose an automatic pest identification method using ViTs which leverages enhanced datasets (through techniques like Histogram Equalization and CLAHE) to avoid overfitting and improve classification accuracy. The ViT network achieved test accuracy that surpassed traditional CNNs by about 1.00%.

Comparing the CNN and Vision Transformer models, certain differences can be observed related to the size of the model, required memory, performance and accuracy.

CNNs are more data-efficient, meaning they tend to perform better on smaller datasets, and usually require fewer computational resources for both training and inference, especially on small to medium-sized models. ViTs often require greater computational and memory resources, especially for high-resolution images. CNNs are well-suited for deployment on Edge devices, and could be used to realize accelerated models because of their lower computational demands and the availability of efficient and compact models.

The choice between CNN and Vision Transformer models may depend on the particular requirements of a given case considering properties such as available resources and data size, as well as the expected compromise between accuracy, model complexity and performance.

CNNs could still have effective applications for many computer vision tasks due to their success across various applications, especially when working with smaller datasets, constrained computational resources, or the need for reliable solutions [57].

Previously presented references indicate the wide possibilities of applying image classification in the field of agriculture, as well as the importance of its execution in real time. Therefore, references related to acceleration models that can be performed on devices near data sources characterized by limited resources are also shown. The example in this paper represents a comprehensive solution that contains algorithm selection, model training, and implementation on end devices. At the same time, the testing environment is defined so that it is possible to straightforwardly implement new classification models using appropriate tools such as Tensil, without the need to know all the details of the FPGA implementation. Also, in such a defined test environment, it is possible to determine a compromise between the type and size of the model on the one hand and the acceptance of accuracy during classification.

## 4. Materials and Methods

### 4.1. Datasets for Training Models

The presentation of our model and concept is given through three applications in the field of smart agriculture. They were chosen because they represent practical examples in image analysis, which involves the classification of images to obtain recognition of current states. Getting information about current conditions in agriculture for these examples could result in optimal usage of pesticide or insecticide. In addition, these applications were selected because there are different datasets in each group collected by the researchers, and we chose one dataset for each application to test the CNN models.

The datasets in the form of images were downloaded from the Kaggle repository. They represent a convenient means of proving the concept of models for image classification. The selected dataset contents are as follows: tomato leaf disease images [58], insect pests [59], and corn weed [60].

The tomato leaf disease images are classified into 10 categories depending on the state of the tomato leaves that could indicate a specific tomato disease or a healthy state [58]. The categories in which a tomato leaf can be found are the following: bacterial spot, early blight, late blight, leaf mold, Septoria leaf spot, spider mites, two-spotted spider mites, target spot, tomato yellow leaf curl virus, tomato mosaic virus, and healthy state. All images were pre-processed and the dimensions of the images used in the training and testing processes were changed to 128 × 128 pixels. This is the first application in our system and is labeled TL-01 (Tomato Leaf-01).

Another application is defined with a CNN model to perform classification on a set of insect pest images [59]. The dataset used contains nine image categories: aphids, armyworm, bollworm, beetle, grasshopper, mosquito, mites, sawfly, and stem borer. The images were preprocessed so that the new image dimensions are 96 × 96 pixels. This application contains the second largest image size and is labeled IP-02 (Insect Pests-02).

The third application relates to the detection of weeds in corn [60]. In this case, there are only two categories, one set of images representing corn and another set of images representing weeds. All images were also pre-processed so that the new image dimension used in the system is 64 × 64 pixels. The third application we used in the system was labeled CW-03 (Corn Weed-03).

From the third set of images, we formed another dataset, where the dimensions of the images would be 32 × 32 pixels. It is an application that has the same properties as the previous CW-03; we just reduced the size of the images in order to check the execution of images on the PYNQ Z2 board (PYNQ is an open-source project of the AMD University Program) for that smaller image format. The application that uses the set of images with the specified reduced format is an additional variant of the third application and we labeled it CW-04.

### 4.2. Tensorflow and Deep Learning on the Edge Devices

TensorFlow is an open-source machine learning framework developed by Google. It provides a comprehensive ecosystem for building and deploying machine learning models, particularly deep learning models. Flexibility is one of the characteristics of TensorFlow 2.12.0 given as it supports both high-level APIs (like Keras 2.12.0) for quick prototyping and low-level APIs for more customization [61].

It is suitable for small models on personal devices as well as large models on distributed computing systems. TensorFlow 2.12.0 can be run on various platforms, including CPUs, GPUs, and TPUs. TensorFlow 2.12.0 offers a wide range of pre-built models, tools, and libraries for various machine learning tasks. It has strong community support with extensive documentation, tutorials, and resources [62].

Deep learning on Edge devices involves deploying machine learning models directly on devices like smartphones, IoT devices, and embedded systems. Edge devices have constraints on memory, processing power, and battery life. Models must be optimized for size and efficiency.

Models for resource-constrained devices could be prepared by model compression with quantization and pruning techniques to obtain smaller model size, using architectures that are based on lightweight models, or training models on devices with limited capabilities.

TensorFlow Lite represents a framework that could be used to optimize models for mobile and embedded devices. It has optimized performance and is designed for efficient execution on devices with limited resources, and has small binary size. It can run on many platforms such as Android, iOS, and embedded Linux devices. It can be used to convert TensorFlow models into a format suitable for mobile or Edge devices. TensorFlow Lite supports GPUs and TPUs for faster inference.

Model compression in TensorFlow Lite involves several techniques to reduce model size and improve efficiency on Edge devices.

Post-Training Quantization is used to convert model weights from 32-bit floating point to lower precision (e.g., 8-bit integers), reducing size and improving inference speed. Quantization-Aware Training is used to train the model with quantization in mind to maintain accuracy.

Pruning techniques remove less important weights, reducing model complexity and size without significantly affecting accuracy.

Weight Clustering is using to group similar weights, reducing the number of unique weights and thus compressing the model [63].

### 4.3. FPGA Acceleration of Deep Learning

FPGAs are programmable hardware devices that can be reconfigured after manufacturing. This flexibility allows them to adapt to different types of computations, including deep learning tasks. FPGAs excel at parallel processing. They can be configured to execute multiple operations simultaneously, making them suitable for tasks that involve large-scale matrix operations and neural network computations.

Developers can design and optimize specific architectures tailored to their deep learning models. This customization can lead to improved performance and efficiency for particular applications. Programming and optimizing FPGAs for deep learning can be complex and requires expertise in hardware design languages (e.g., Verilog, VHDL) and tools (e.g., Xilinx Vivado, Intel Quartus).

FPGAs are also used in data centers to accelerate specific workloads and tasks, offering flexibility in adapting to changing computational demands. Also, FPGAs can be deployed in Edge devices for on-device processing of deep learning models, enhancing privacy and reducing latency by avoiding round-trip data transfer to Cloud servers. FPGAs can offer low latency and high throughput when properly configured for specific tasks, making them suitable for real-time applications in Edge computing scenarios [64].

Xilinx Vivado is a comprehensive development environment and toolchain specifically designed for programming and optimizing Xilinx FPGAs. Xilinx Vivadouses RTL (Register Transfer Level) Design, which means it supports hardware description languages like Verilog, VHDL, and SystemVerilog for specifying digital circuits.

Also, High-Level Synthesis (HLS) could be used to convert C/C++ code into RTL for FPGA implementation.

Xilinx Vivado has the ability to run simulations that perform functional verification of the FPGA design before synthesis. It ensures that the design meets timing requirements and constraints, and provides debugging capabilities to identify and resolve issues in the FPGA design. It allows developers to customize FPGA designs at a low level, optimizing performance for specific applications and facilitates integration into complex systems through a comprehensive toolchain and IP core library.

Xilinx Vivado could be used for designing custom hardware accelerators for specific algorithms or applications, including deep learning inference. Xilinx Vivado includes a library of pre-designed Intellectual Property (IP) cores for common functions, simplifying design reuse and accelerating development [65].

### 4.4. Residual Network Based on CNN

A Residual network (ResNet) is a type of deep neural network that introduces skip connections or shortcuts to address the problem of vanishing gradients in very deep networks. It can be obtained from the CNN network by adding the input of the previous layer to the output of the current layer. This allows the network to learn more effectively and can enable better performance. ResNets were first introduced by Kaiming He et al. [66] with results that won the Best Paper Award at the 2015 Conference on Computer Vision and Pattern Recognition (CVPR).

A residual block consists of a few convolutional layers, typically two or three, along with a shortcut or skip connection that bypasses these layers. The output of the convolutional layers is added to the original input of the block (the identity connection), forming the block’s final output.

Skip connections help to mitigate the vanishing gradient problem by allowing gradients to flow directly through the network during backpropagation, thus improving the training of deep networks. Skip connections enable the network to learn identity mappings, making it easier to optimize and allowing for the training of much deeper networks. The architecture of a typical ResNet consists of multiple residual blocks stacked together, along with initial convolutional and pooling layers and final fully connected layers. They have become a foundational architecture in deep learning and have significantly advanced the field of computer vision [67].

The authors of [68] present the theoretical foundations of the CNN concept and architecture. Table 1 summarizes popular CNN architectures with important characteristics indicated.

In the table, among the prominent types of CNNs, there is a ResNet architecture with its advantages, which was selected for the implementation of our model. Since it represents the concept of residual learning, it positively affects the difficulty of network convergence. In this way, learning ability and performance in the image classification process are improved. Specifically, ResNet20 with a small number of layers was used, since the intention was to transfer the model to devices with limited resources within Fog computing.

### 4.5. Tensil AI

Tensil is a set of tools that provides hardware and software solutions for accelerating machine learning models, particularly for Edge devices. Tensil aims to offer efficient, scalable, and high-performance machine learning inference through its specialized hardware and software stack.

Tensil offers AI accelerators that are customizable and can be tailored to meet specific performance, power, and area requirements. These accelerators are designed to be integrated into System-on-Chips (SoCs) for various Edge applications.

The architecture of Tensil’s solutions is scalable, meaning it can be adapted to various performance levels depending on the application requirements. This scalability ensures that a wide range of devices, from low-power IoT sensors to high-performance Edge servers, can benefit from their technology. Designed for low power consumption, Tensil’s hardware accelerators are ideal for battery-operated devices and applications where energy efficiency is critical.

Tensil provides a comprehensive software stack that includes tools for model optimization, deployment, and management. This software stack helps developers convert their trained machine learning models into formats that can run efficiently on hardware [69].

### 4.6. PYNQ Z2 Description and Characteristics

The PYNQ Z2 is a development board that combines the power of a Xilinx Zynq-7000 SoC with the flexibility of Python programming. The board features a Xilinx Zynq-7000 SoC, which includes a dual-core ARM Cortex-A9 processor and an FPGA fabric. This combination allows for both high-level software processing and customizable hardware acceleration.

The PYNQ Z2 board offers a range of features and characteristics that make it suitable for various applications. It has 512 MB of DDR3 memory, which provides ample space for running complex algorithms and storing data. The board also includes 16 MB of Quad-SPI flash memory for storing boot files and other essential data.

**Figure 2:**
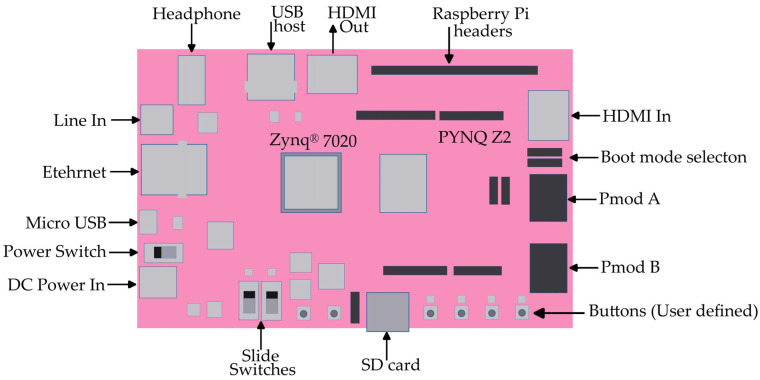
PYNQ Z2 board.

In terms of connectivity, the PYNQ Z2 offers a variety of options. It has HDMI input and output ports, allowing for easy integration with displays. It also includes USB ports, Ethernet, and Wi-Fi capabilities, enabling communication with other devices and networks (Figure 2). Additionally, the board has Arduino and Raspberry Pi headers, providing compatibility with a wide range of expansion modules and accessories [70].

One of the standout features of the PYNQ Z2 is its support for the PYNQ (Python Productivity for Zynq) framework. This framework allows developers to leverage the power of Python and the flexibility of the FPGA fabric to accelerate their applications. With PYNQ, developers can easily program the FPGA using Python libraries and take advantage of hardware acceleration for computationally intensive tasks.

Its features and characteristics make it suitable for a wide range of applications, from embedded systems development to digital design prototyping. The ARM cores and FPGA fabric can communicate with each other through a high-bandwidth interconnect, enabling efficient data exchange and collaboration between the software and hardware components of a system [71].

### 4.7. Working and Testing Environment

**Figure 3:**
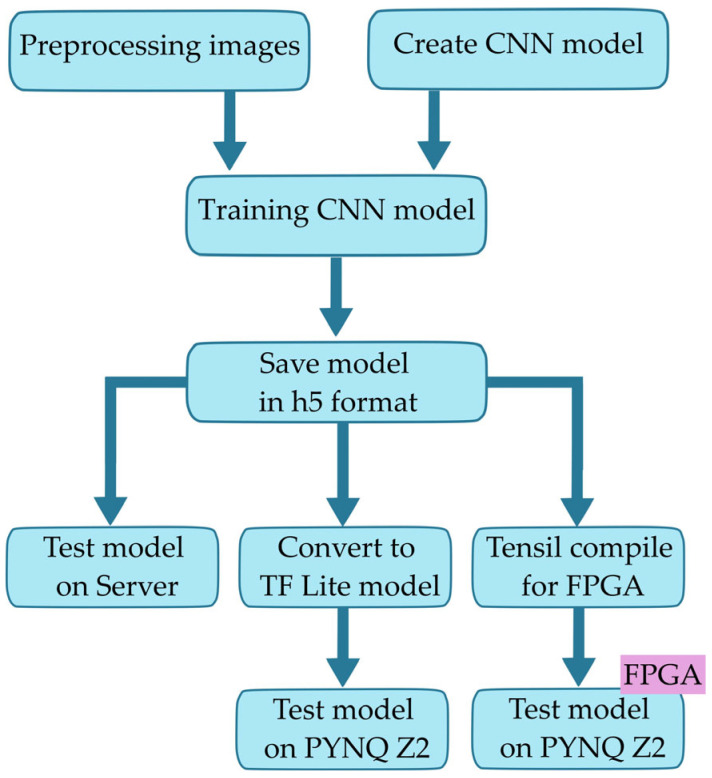
Training CNN models and preparing for image classification on the server and PYNQ Z2.

The entire environment where the classification model was created and trained, as well as tested, was placed on the server. After that, the trained models were converted into optimized models for running on resource-constrained devices. The entire process of preparing the CNN model is shown in Figure 3. First, the images were pre-processed in order to obtain the appropriate dimensions intended for model training and validation. A CNN model was created using ResNet 20 and all necessary parameters were defined in order to perform the training process as efficiently as possible. After achieving satisfactory accuracy, the given CNN is saved in h5 format for further use. At that moment, it can be immediately tested on the server with the help of test data that were not used for training.

The given model in h5 format is loaded and its conversion into TensorFlow lite model is performed. Also, the same model is converted with the help of the Tensil framework to the model format that would be intended to be executed on FPGA. These new forms of models as well as test data are transferred to PYNQ Z2 where they are run.

**Figure 4:**
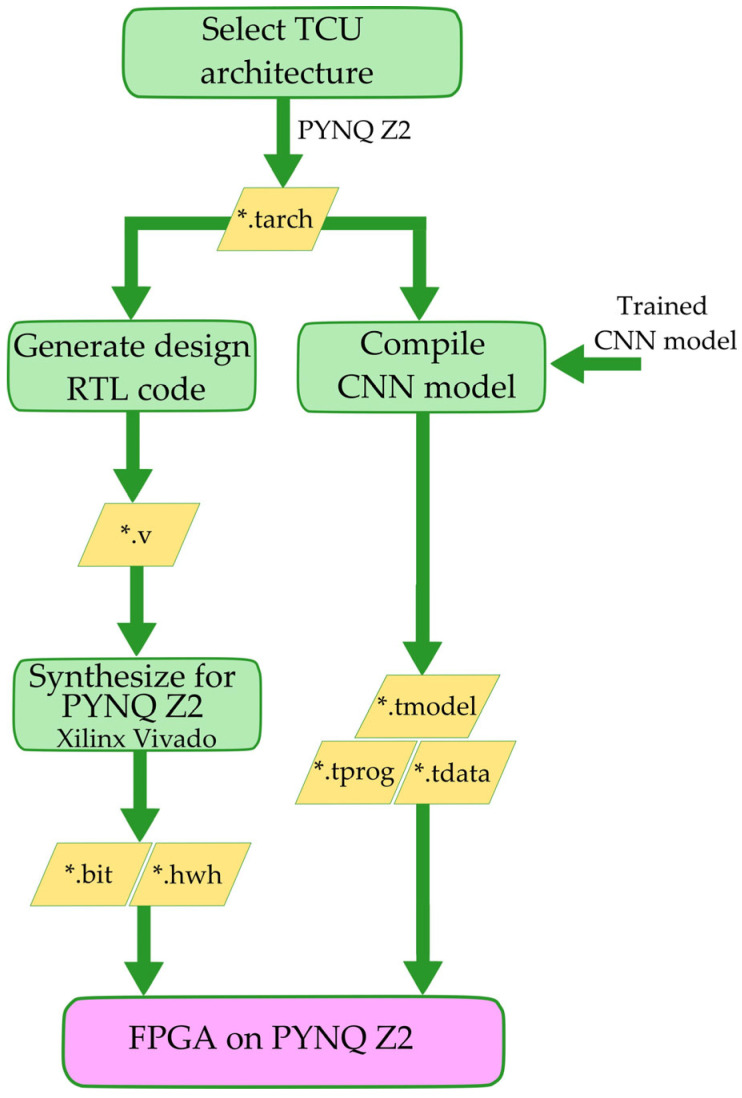
Preparation of CNN models to run on PYNQ Z2.

The process of converting a CNN model with the help of the Tensil framework and preparing for its execution on FPGA is shown in Figure 4.

First, the PYNQ Z2 architecture was selected, which is given as records in a file with extension “tarch”. Then, the same architecture file was used in two processes by the Tensil framework. First, the design was generated with the help of Tensil, which produced the Verilog files. These files are loaded into the Xilinx Vivado, where the preparation and synthesis of the project is carried out, which enables the execution of the CNN model on the FPGA. On the other hand, with the help of the selected architecture, the pretrained CNN model is converted, resulting in three files: *.tmodel, *.tprog and *.tdata. All these files are transferred to PYNQ Z2 and image classification is called from the Python environment using the CNN model which is now executed on the FPGA.

**Figure 5:**
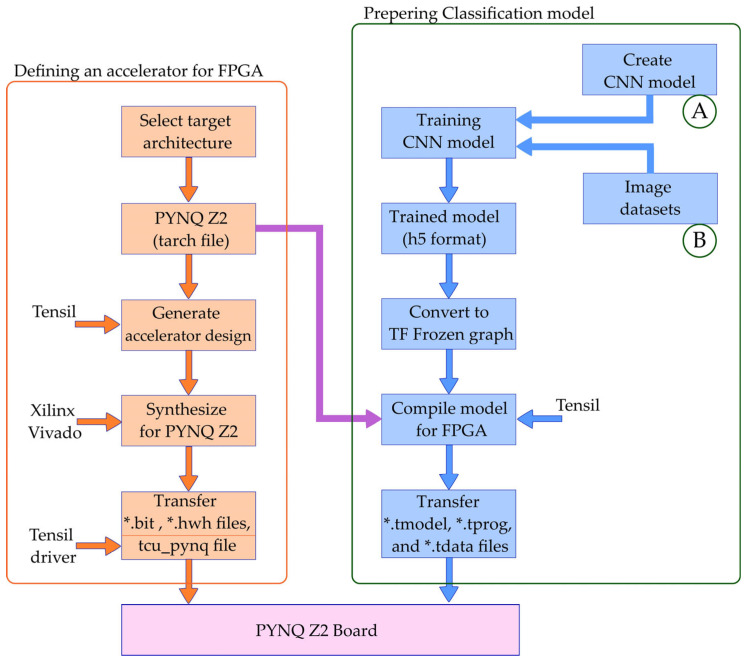
Preparing an acceleration model for image classification on FPGA.

The entire process of preparing a classification system from model selection and training to the server and its compilation and transfer to a device with limited resources, as a model accelerator on the FPGA, is shown in Figure 5. In order to simplify the implementation and application of the model, the process is divided into two branches, the resulting files of which are transferred to the PYNQ Z2 board, where the image classification is performed. The first branch represents the selection of an architecture, which in this case is PYNQ Z2, then the generation of the accelerator design, its preparation and synthesis with the help of Xilinx Vivado. Finally, the resulting bit file and the driver file are used on the PYNQ Z2. In the case of choosing and using this particular architecture, the procedures described are carried out once and the resulting files can be reused for different classification models that are executed on this hardware.

The second branch is not completely independent from the first, but it contains all the steps for choosing a classification model and preparing it for execution on the FPGA. Dependency to the first part is reflected in the fact that when compiling the pre-trained model with the help of Tensil [69], the chosen architecture is taken into account, which can be clearly seen from Figure 5. The steps in the second branch are repeated every time when a new model is prepared for image classification. The approach formed in this way implies that it is very simple to repeat the process for any new model. The only thing that needs to be included is a new classification model (A) and input datasets with a suitable set of images (B). All the other processes below are repeated and thus the classification model for the FPGA is effectively obtained without the need to know the details of the FPGA implementation.

## 5. Results and Discussion

**Figure 6:**
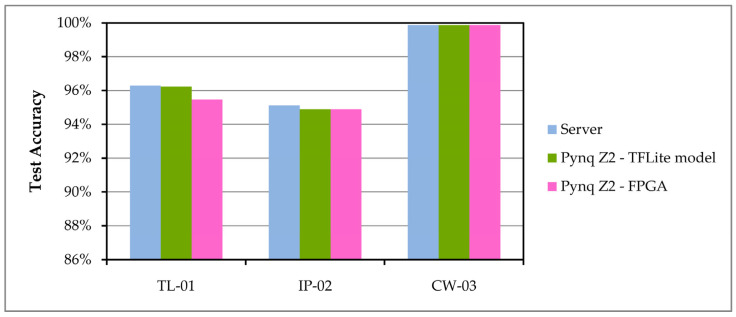
Test accuracy for CNN models run on server and PYNQ Z2.

The testing of CNN models, which are used in the three implemented applications, was performed on new datasets that were not used during training. Three tests were performed for each model, one test on the server with the original model and two tests on PYNQ Z2 with the converted CNN model for devices with limited resources. The test accuracy values are shown in Figure 6, where it can be seen that there was no significant decrease in accuracy after converting and using the model on the Fog computing layer.

The achieved test accuracy when using the classification models has quite high values, which can be seen from Figure 6, while in the third application in our example, the accuracy even reached 99%. Such a high value was achieved because in the third application (CW-03) there are only two classes, allowing one of the two possible variants to be recognized very successfully on a given set of images.

After testing the CNN models, we proceeded to check the performance of the system depending on where the data is processed. We formed a test dataset of images and sent one image at a time to the classifier module. We calculated the time elapsed from the moment of sending the image to the moment when the result arrives in the form of the category to which the image belongs.

**Figure 7:**
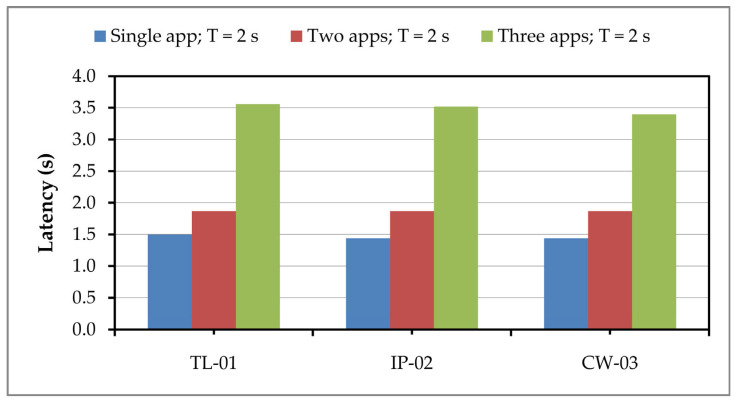
Latency in image classification on the server for different application settings.

There were three different main variants regarding the type where the classification process is carried out. In the first variant, we sent one image at a time to the server via the MQTT protocol, where the receiving model downloaded the image and performed classification with the help of the pre-trained CNN model. After that, using the MQTT protocol, the response would be returned to the device that sent the request. The elapsed time or delay for receiving a response from the server in case of all three applications and their combinations is presented in Figure 7.

When only one application is running, it can be seen that the delay is about 1.5 s. We chose in our test examples that the first period of sending images should be 2 s (T = 2 s). If the images were continuously sent in a time interval of less than 1.5 s in this setting, the time required for the classification process would accumulate and lengthy delay times would be obtained, which would prevent the expected system functionality. When two applications are running, a slightly higher delay value is obtained, but still below the sending period of 2 s. If all three applications on the server are started, and receive images periodically from the Fog device, then their delay increases to a value of about 3.5 s. A further increase in data transfer or additional network activity on the server can cause a higher value of latency, which leads to a situation that we do not expect from a system where getting a quick response is very important.

The idea of transferring the classification model to the level of Fog computing was demonstrated in order to improve the reliability of the application, among other things. If the model were executed on the server, the amount of data would affect the response time so that in the case of a larger number of running applications or due to an increase in the frequency of sending data, the delay time would be significantly increased. Under such conditions, the delay would be high, so that it would jeopardize the normal functioning of the system. If the classification model were executed on a Fog device close to the data source, the response would be obtained in real time and the reliability of the system would not depend on a constant connection to the Cloud and network load.

**Figure 8:**
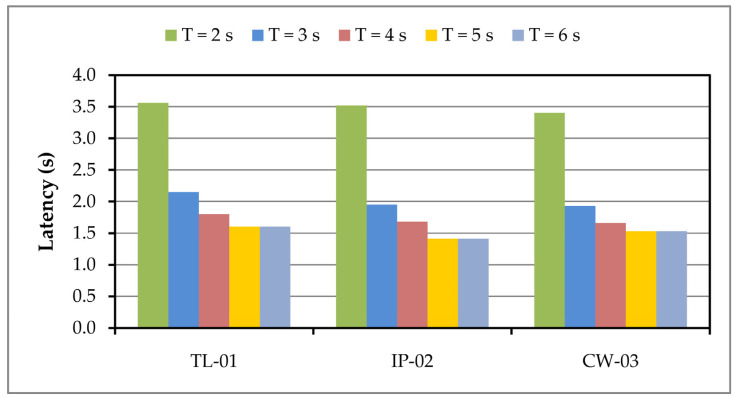
Latency in image classification on the server running all three applications.

If in the variant with all three applications we increase the period of sending data, i.e., the interval between sending, then the load on the server decreases and thus the value of the delay (Figure 8).

When the image sending period is increased to 5 s, as well as to 6 s, in a given system setting, the delay is reduced to near 1.5 s, setting it to the value it had in the case of individual applications.

**Figure 9:**
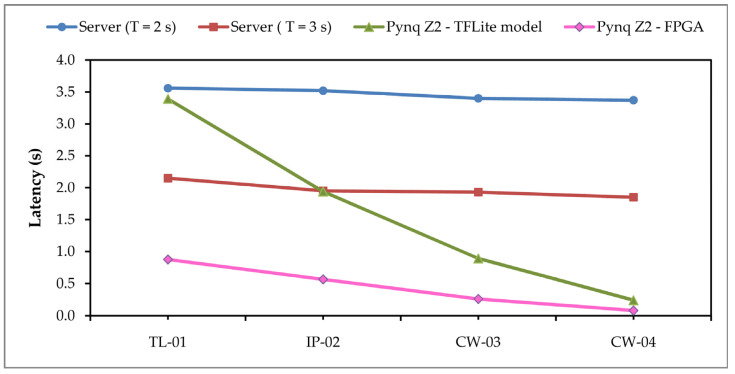
Time elapsed in receiving result of image classification.

Apart from the classification of images on the server, the other two variants refer to the processing of data on the PYNQ Z2, so that the images are forwarded to the TensorFlow Lite model and the FPGA part of the device for the execution of the classification process. The time required for image classification (latency) in the case of execution on the server as well as on the Fog level via PYNQ Z2 is presented in Figure 9.

In this graph, the same server latency data for the T = 2 s and T = 3 s periods as in the previous figure can be seen, but now in relation to the latency values of the PYNQ Z2. As expected, the delay is lower on the Fog computing layer, but the values are not the same for all three application variants. For the first application, TL-01, classification is performed on images that have the largest dimension of 128 × 128 pixels, and therefore during testing we had the largest delay. In the second application, IP-02, images with dimensions of 96 × 96 pixels were used and, accordingly, a lower delay was obtained. The third application, CW-03, accepts and classifies images with dimensions of 64 × 64 pixels and accordingly there is a delay of 0.89 s for the TF Lite model and 0.26 s for the FPGA classification. Taking these delay values, the number of images to be classified per second can be determined, which for the CW-03 application is 3.85 images per second. In Figure 8, we also present an application labeled CW-04, which represents the same application as CW-03, only with different inputs, since it uses images with dimensions of 32 × 32 pixels. In this variant, even less delay time is obtained, and a significant acceleration in image processing via FPGA is achieved.

One of the datasets we used to demonstrate the presented image classification system concept is a set of tomato leaf disease images, which correspond to images from the Plant Village dataset [72]. A dataset related specifically to images of tomato leaves has been used by various authors in experiments to classify tomato diseases. Various algorithms were used and the results of the applied models expressed by accuracy are shown in Table 2. The table contains several studies that represent the basis for comparison in terms of model accuracy [55]. Each row contains the reference of the author’s research, the dataset used, the algorithm and the testing accuracy, which can be compared with the last two rows in the table that contain the description of the model presented in this paper.

Although the presented model has its compact size with the aim of being placed on devices with limited resources, its accuracy still has a high value. After training the model on the server, it was tested with unused data and a testing accuracy of 96.29% was achieved. After the model was optimized and translated to a version of the model that can be run on the FPGA, testing was performed with the same new data and a test accuracy of 95.46% was obtained. Although the model was adapted and compiled for FPGA, the given testing accuracy was very slightly reduced compared to the original trained model on the server. Compared to the results from other articles, it can be seen that the accuracy of the proposed model for classification is acceptable even in the case of preparing its version for FPGAs within the concept of Fog computing that characterizes devices with limited resources.

Precisely because of the cases with limited resources, in order to meet the requirements of the concept of Edge or Fog computing, the image classification algorithm and other parameters were selected in order to obtain a model with a smaller and more compact size. In Table 3, various deep learning models are presented [54], with the number of their model parameters, model size and number of frames per second on ARM Cortex processor.

According to the data in the table, it can be seen that the proposed model for classification has a far smaller number of parameters than other models, and also a smaller size. Another parameter that shows the performance of the model is the execution time, in this case the number of images per second, on a device with an ARM Cortex processor, and this is presented in the last column. For our model in the last two lines we present two variants depending on the size of the input data. In the case of classification of images with dimensions of 128 × 128 pixels, processing of 0.3 images per second is achieved on an end device with limited resources. If we used a set of smaller images, 32 × 32 pixels, then the image processing value would be 4.17 images per second. The stated values in relation to the other models fit into their range, even though ours is a much smaller model.

Table 4 shows the hardware platforms that can make up the Edge devices on which the classification model will be run. Certain platforms were selected based on the comparison presented in article [80], with the platform used in our work for FPGA acceleration entered as the last row in the table for comparison.

Especially when considering the execution values of the same model compiled for FPGA, improvements can be seen. Testing the accelerated model on FPGA, we get results (Figure 9) that for the first TL-01 application, the number of images per second is 1.14, while in the second variant for the CW-04 application, the number of images per second is 12.2.

**Figure 10:**
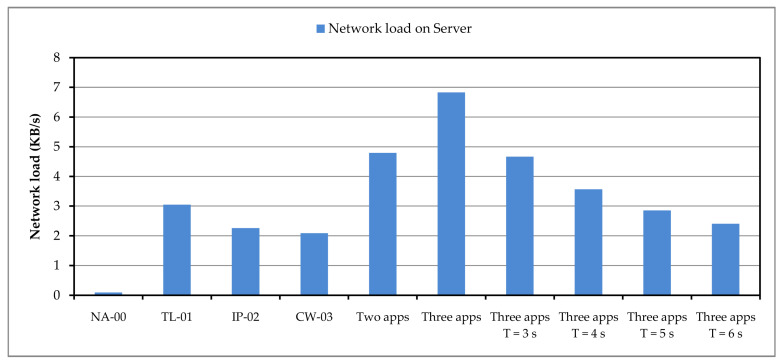
Network data transfer to the server.

Processing data on the Fog computing layer and getting fast and reliable responses is beneficial in itself, but it also saves resources on the server side. The images from the end devices are not sent to the remote server and therefore the data transfer over the network is lower, especially if there are a large number of devices. In the case of our system setup, the network load for different variants of the three selected applications (TL-01, IP-02 and CW-03) is shown in Figure 10.

Within the defined system settings, the influence of running applications on the network load at the server was measured. During the testing, the network data were captured and their average values are shown in Figure 10, where NA-00 represents the network load when no applications are running. The next three values represent the amounts of network data in the case of individual applications, according to the size of the data transferred for each application. A higher value is obtained when two applications are running and the highest network traffic value is for all three applications. The tests described were carried out with a period of 2 s (T = 2 s), and if the period of sending images to the server were to be increased (3, 4, 5, or 6 s), the network data transfer on the server would be reduced accordingly.

**Figure 11:**
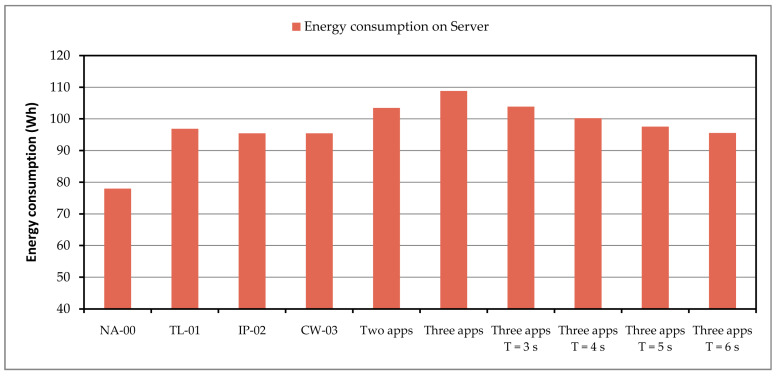
Energy consumption on the server during application testing.

In the case where most of the data stream processing is transferred to the Fog computing layer, energy consumption on the server is also saved. The values of energy consumption on the server, during testing, observed on average for one hour, are presented in Figure 11.

The value labeled with NA-00 represents the consumption value when none of the test applications are started; the server only has its other services active, configured according to the previously defined system settings. Similar to the previous graph, the highest consumption is in the case where all three applications are running. If the period of sending data to the server increases, the intervals when the classification process is performed are longer, and then the value of the energy required for executing applications is reduced.

The presented results of the test system show the relationships between important parameters in the case of executing applications in the Cloud or transferring them to the level of Fog computing. The test system not only contains the part related to the training of CNN models, but also support for their preparation and transmission at the Fog computing level.

Realized applications in smart agriculture are also accomplished through CNN image classification that can be performed on the Fog devices. Therefore, user applications can be defined in such a way that the classification results could be obtained in real time. Such benefits enable further application development in the field of smart agriculture by providing the opportunity to optimize CNN models and their execution on devices with limited resources close to the location of the data source.

The entire test system with the parameters obtained during application testing can be used to consider the benefits of using Cloud-Fog computing. Also, the existing structure of the test system can be transferred to another environment that has completely different configuration and resources. In this situation, the same types of parameters could be obtained quite effectively, performing tests of user applications, and providing indicators to evaluate a new Fog computing system.

## 6. Conclusions

Image analysis using a classification process provides recognition of various conditions in the field of agriculture which enables many resource savings, optimization and timely reactions. In this article, we have presented a working setup for creating, training and using image classification models in the domain of smart agriculture. This realization of the classification model represents a comprehensive solution, from data preparation, through defining the structure of the neural network, and the training process, to the preparation and implementation of the model on devices that belong to the level of Fog computing. The transfer of image classification near the data source to devices with constrained resources allows a significant contribution in smart agriculture applications as it enables real-time response, operational reliability and independence from constant data transfer to the Cloud. Also, the test part of our setup can be used to check the performance of new configurations or new solutions. By running tests on other configurations, it would be possible to determine what the delays would be, and the network load depending on the number of application instances and the frequency of sending data. Also, our test setup could be used for determination of device potential at the Fog computing layer for executing selected IoT applications. Future research directions could be related to the application of transfer learning techniques to address the challenges of limited datasets in agriculture. A significant insight would be to examine the effectiveness of applying pre-trained models for data classification and transfer learning in the case of small datasets. The classification models employed were related to the applications used for recognition of weeds, insect pests and tomato leaf diseases, and could contribute to the reduction of sensitive substances appliance, such as fungicides and insecticides.

## Figures and Tables

**Table 1:** Popular CNN architectures and their comparison.

Architecture	Year	Highlights	Strength
LeNet	1998	Rapidly deployable and effective at resolving small-scale image recognition issues.	Utilized spatial correlation to decrease computation and parameter count.
LeNet-5			Automated discovery of feature hierarchy structures.
AlexNet	2012	AlexNet is comparable to LeNet-5, except it is more complex, has more filters per layer and employs stacked convolutional layers.	Low, middle, and high-level feature extraction utilizing large and tiny size filters on the early (5 × 5 and 11 × 11) and final (5 × 5 and 11 × 11) layers (3 × 3).
			Implemented regularization in CNN. Commenced parallel usage of GPUs as an accelerator to address difficult architectures.
ZfNet	2014	Conceptualization of middle levels.	Illustrated parameter tweaking by displaying the output of intermediary layers. Diminished the filter size and stride in the initial two layers of AlexNet.
VGG	2014	The accuracy of a model is improved by employing small convolutional filters with dimensions of 3 × 3 in each layer.	Introduced the concept of an effective receptive field. Presented the concept of a simple and homogeneous topology.
GoogLeNet	2015	A deeper and broader architecture with various receptive field sizes and a number of extremely small convolutions.	Introduced the concept of applying multiscale filters to layers. Introduced the concept of divide, transform, and merge. Reduced the number of parameters by the use of bottleneck layer, global average-pooling at the final layer, and sparse connections. Use of auxiliary classifiers to enhance convergence rate.
Inception-V3	2015	Enhances the efficiency of a network. The application of batch normalization expedites the training process. Inception-building elements are employed effectively to go deeper.	Utilized asymmetric filters and bottleneck layer to decrease the computational expense of deep designs.
ResNet	2016	A unique design that features “skip connections” and extensive batch normalization.	Reduces the error rate of deeper networks; introduces the concept of residual learning; mitigates the vanishing gradient problem.
DenseNet	2017	All layers are intimately connected to one another in a feed-forward fashion. It mitigates the problem of vanishing gradients and requires few parameters.	Added depth or cross-layer dimension. Ensures maximum data flow across network layers. Prevents relearning redundant feature-maps. Both low-level and high-level features are available to decision layers.
DenseNet-121			

**Table 2:** Comparison of testing accuracy for tomato diseases classification.

No.	Articles	Tomato Dataset	Algorithm/Model	Testing Accuracy
1	Abbas et al. [73]	Plant Village Synthetic	DenseNet121	97.11%
2	Hossain et al. [74]	Plant Village	Multi-Axis Vision Transformer	93.00%
3	Agarwal et al. [75]	Plant Village	VGG16	91.20%
4	Barman et al. [55]	Plant Village	Vision Transformer	90.99%
5	Proposed Work—on server	Part of Plant Village dataset	ResNet20	96.29%
6	Proposed Work—on FPGA accelerator	Part of Plant Village dataset	ResNet20	95.46%

**Table 3:** Parameters comparison of proposed classification system and other DL models.

Model	Parameters (10^6^)	Model Size (MB)	FPS (ARM Cortex Processor)
VGG19 [76]	200.25	229.0	0.47
VGG16 [76]	147.15	168.0	0.62
EfficientNetB0 [77]	4.05	46.9	2.69
MobileNetV1 [78]	3.23	37.1	8.23
MobileNetV3Small [79]	1.53	18.0	7.43
Vit Base [48]	86.00	345.0	0.21
GreenViT [54]	21.65	247.0	0.34
Proposed Work (TL-01 app)	0.29	3.8	0.30
Proposed Work (CW-04 app)			4.17

**Table 4:** Hardware platforms as Edge devices.

Hardware Platform	Processing Unit	AI Acceleration	Memory	FPS
Tinker Edge R	ARM Dual-core Cortex A72, Quad-core cortex A53	NPU	4 GB LPDDR4, 2GB LPDDR3	6.5
Raspberry Pi 4	ARM Quad-core Cortex A72	-	4 GB LPDDR4	4.8
Google Coral	ARM Dual-core Cortex A53	TPU	1 GB LPDDR4	3.6
NVIDIA Jetson Nano	ARM Cortex A57	128-core GPU	4 GB LPDDR4	7.2
Proposed Work with PYNQ Z2 (CW-04 app)	ARM Dual-core Cortex A9	Programmable Logic (FPGA)	512 MB DDR3	12.2

## Data Availability

The original contributions presented in the study are included in the article, further inquiries can be directed to the corresponding authors.
